# Neural signatures of extreme sensitivities to light: cortical markers in hypersensitive and hyposensitive individuals via EEG

**DOI:** 10.3389/fnins.2025.1542154

**Published:** 2025-03-10

**Authors:** Valerio Salvati, Satoru Otani, Elisa M. Tartaglia

**Affiliations:** ^1^Institut national de la santé et de la recherche médicale (INSERM), Centre national de la recherche scientifique (CNRS), Institut de la Vision, Sorbonne Université, Paris, France; ^2^Center of Innovation and Technologies Europe, Essilor International, Société par actions simplifiée (SAS), Paris, France

**Keywords:** light, sensitivity, EEG, photophobia, discomfort, hypersensitive, hyposensitive, hyperexcitability

## Abstract

Light plays a crucial role in human biology. However, while the general pathways involved in light perception are well-understood, the specific neural mechanisms explaining why some individuals experience an adverse behavioral response to light (hypersensitivity), while others rather the opposite (hyposensitivity) remain unclear. Here, leveraging the high temporal resolution of EEG, we set out to test the hypothesis that, in hyposensitive individuals, an excessive sensory stimulation may lead to neural hyper-excitability. Such an enhanced response, in turn, might be key to mitigate discomfort. We conducted our study on 21 participants, who underwent light exposure tests at varying intensities. Our findings revealed that hyposensitive individuals, who are less averse to intense light exposure, can rely on a more efficient neuroprotective mechanism against sensory overload, when compared to hypersensitive individuals. Such a mechanism is mainly and consistently expressed through the increase in power of beta and gamma oscillations, along with a delayed onset of the P100 component in response to light stimuli. These findings open the door for future research to adaptive technologies that utilize EEG markers to create personalized, real-time interventions for light sensitivity, such as adaptive wearable devices or environmental systems that dynamically adjust lighting based on neural feedback, providing immediate relief for hypersensitive individuals.

## 1 Introduction

As the primary source of visual information, light influences biological rhythms (Czeisler and Gooley, 2007; Chang et al., 2015), cognitive processes (Vandewalle et al., 2009; Chang et al., 2015), and emotional states (Bedrosian and Nelson, 2017; Chang et al., 2015; Knez, 2001). It is central to both ocular and neural systems, and its impact extends beyond perception, influencing behaviors, social practices, and even art throughout human history (Fabiano, 2023).

Humans exhibit an extraordinary sensitivity to light, with the human visual system capable of detecting even a single photon captured by a rod photoreceptor, although, on average, it takes 5-8 photons to perceive a flash of light due to intrinsic noise in the visual system (Lakshminarayanan, 2005). However, not all individuals share the same threshold for light perception. For example, some individuals are hypersensitive to light, experiencing discomfort or adverse effects even under normal lighting conditions. On the other hand, hyposensitive individuals may require stronger light stimuli to react similarly. Moreover, different sensitivities to light also manifests in behavioral and cognitive changes. Hypersensitive individuals may struggle with prolonged exposure to light, which can lead to discomfort, headaches, and even migraines, while hyposensitive individuals may not show such immediate reactions but may suffer from long-term consequences of insufficient light exposure, such as disrupted circadian rhythms and reduced cognitive alertness (Bourgin and Hubbard, 2016).

More rigorously, hypersensitivity (photophobia), refers to an abnormal or heightened sensitivity to light, typically manifesting as discomfort or pain when exposed to normal or bright light conditions (discomfort glare). From an ocular perspective, photophobia is often associated with dysfunctions or irregularities in the structures responsible for detecting and processing light. These include the cornea, lens, and retina, where light is converted into electrical signals. Conditions such as dry eye, corneal abrasions, uveitis, or cataracts can make the eyes hypersensitive to light by affecting this process (Digre and Brennan, 2012). Intrinsically photosensitive retinal ganglion cells expressing melanopsin (ipRGCs) have also been identified as contributors to the regulation of non-image-forming visual functions such as circadian rhythm and pupillary light reflex, which can influence light sensitivity (Do, 2019). The trigeminal nerve, which is responsible for facial sensation, also plays a role in ocular light sensitivity. When stimulated excessively by light, the trigeminal pathway can contribute to the sensation of pain or discomfort experienced in photophobia. This is especially significant in patients with migraines, where over-sensitization of this pathway is a known trigger for light-induced headaches (Noseda et al., 2010). Differences in sensitivity to light can be attributed to genetic factors, such as the density of the macular pigment and lens, cone cell sensitivity, and the L/M cone ratio. For example, a study on sex differences in sensitivity to light found that men respond more strongly to blue-enriched light in the evening compared to women, with faster reaction times and higher brightness perception (Chellappa et al., 2017).

Although ocular components are crucial in initiating light perception, the processing and modulation of these signals within the brain play a vital role in the development of photophobia. Studies have shown that abnormal brain activity in regions such as the thalamus, visual cortex, and the brainstem can exacerbate light sensitivity. Specifically, the thalamus serves as a sensory relay hub that processes light signals before they are sent to other areas of the brain, and alterations in thalamic function can result in misregulated responses to light (Younis et al., 2019). Additionally, brainstem pathways associated with migraine pathology, such as the trigemino-vascular system, may be hyper-excitable in individuals with photophobia. This heightened response is evident in imaging studies of both healthy subjects (Bargary et al., 2015) and migraine patients, where abnormal activity in the visual cortex correlates with increased sensitivity to light (Burstein et al., 2000).

In neurodegenerative diseases such as multiple sclerosis, patients frequently experience photophobia due to optic nerve inflammation (optic neuritis) and impaired transmission of visual information, further illustrating the interconnectedness of neural and ocular mechanisms underlying light sensitivity (Cortese et al., 2018).

Previous research seems consistent in proposing that an intense light stimulation triggers a hyper-excitability of the neural activity (Bargary et al., 2015), associated with a negative behavioral response indicating discomfort. Here, we propose that hyper-excitability represents a protective response to an overwhelming sensory stimulation, and hence the increase in activity protects the system from the incoming light. We used EEG to capture the dynamics of the cortical markers associated to both hyposensitive and hypersensitive individuals. According to our hypothesis, we expect the former to be able to deploy a stronger protective mechanism against discomforting incoming light, and hence some form of heightened neural activity, with respect to the latter. To test our hypothesis, we analyzed both the temporal and spectral characteristics of the neural responses to light stimuli in both populations. Our results are based on two main types of analysis: a general population analysis and a comparison between hypersensitive and hyposensitive individuals. The general population analysis aimed to identify common cortical responses to different light intensities, while the group comparisons sought to highlight neural differences between those with higher and lower sensitivity to light.

## 2 Materials and methods

### 2.1 Participants

A total of 21 adult participants, who were not affected by any eye or brain pathology, took part in the study (mean age 34.5, std 9.4). This study involving human participants was reviewed and approved by Comite de Protection des Personnes Ile de France III Hopital Tarnier-Cochin, Paris). Participants provided their written informed consent to participate in this study. No participants reported being under the influence of any substance that could impact the outcome of the test. Thorough explanations about the purpose of the study and the specific procedures involved were provided to all participants, according to the principles outlined in the Declaration of Helsinki. Subsequently, written consent was obtained from each participant, ensuring their informed agreement to participate in the research.

### 2.2 Stimuli and set-up

**Figure 1:**
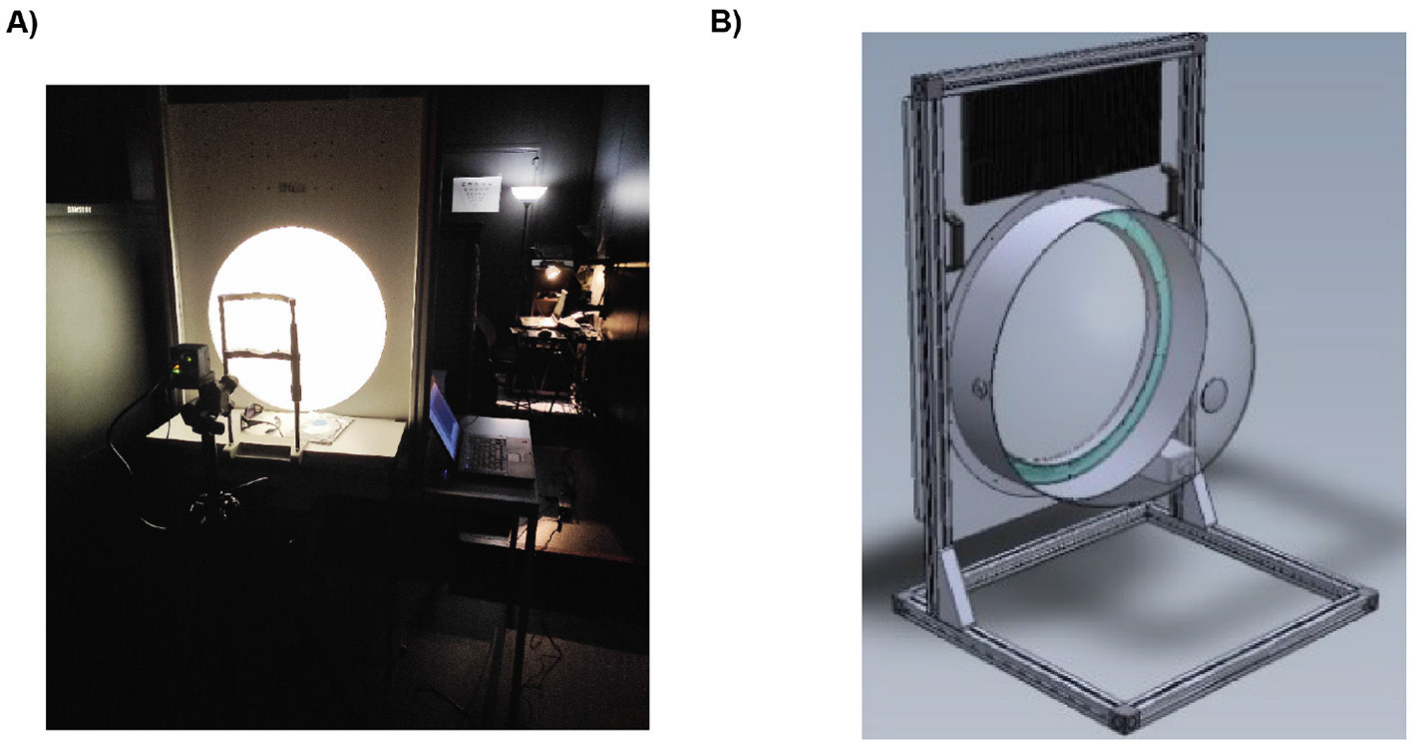
**(A)** The EFFILUX dome projecting light in the experimental room. **(B)** 3D sketched model of the dome. In green the position where the LEDs are located.

We used a custom Effilux Illumination Dome (EFFI-Dome-700-WarmWhite, modified by Effilux according to our specifications) to create a controlled and immersive lighting environment (Figures 1A, B). The dome has a diameter of 70 cm and is fitted with an array of warm-white LEDs. These LEDs can deliver a maximum illuminance of 24,800 Lux on the surface of the dome. The system offers an illuminance resolution of 30 Lux, enabling precise adjustments to the light intensity. A notable feature of the Effilux Dome is its ability to produce uniform illumination through the reflection of light from the LEDs. The dome is connected to a computer via USB, allowing the execution of a script that controls the modulation of light intensity and duration. During the experiment, participants sat in an armchair in front of the dome with their heads supported by a chinrest. The surrounding room was dimly lit, and participants were instructed to fixate in the center of the dome with both eyes open.

### 2.3 Protocol

**Figure 2:**
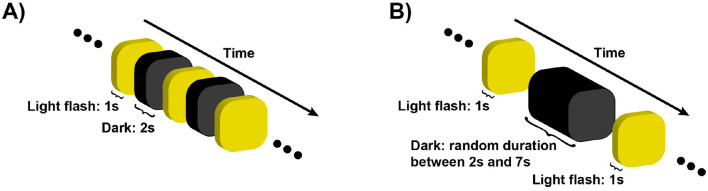
The sketch illustrates the temporal dynamics of the behavioral assessment **(A)** and the EEG protocol **(B)**. In the behavioral assessment **(A)**, light flashes of increasing intensity were projected for 1 s, followed by a 2 s dark period. Participants pressed a button once to indicate the onset of discomfort due to the light intensity (*i*_2_), and a second time when the discomfort became unbearable (*i*_4_), at which point the assessment was stopped. **(B)** shows the EEG protocol, where personalized light intensities (*i*_1, 2, 3, 4_), determined from the behavioral assessment, were projected randomly shuffled for 1 s, interleaved with dark periods of random durations ranging from 2 to 7 s. The randomization of both the light intensities and the dark intervals was carefully designed to prevent the brain and eyes from predicting the timing of the flashes. In total, 200 trials were conducted, consisting of 50 trials for each condition.

**Figure 3:**
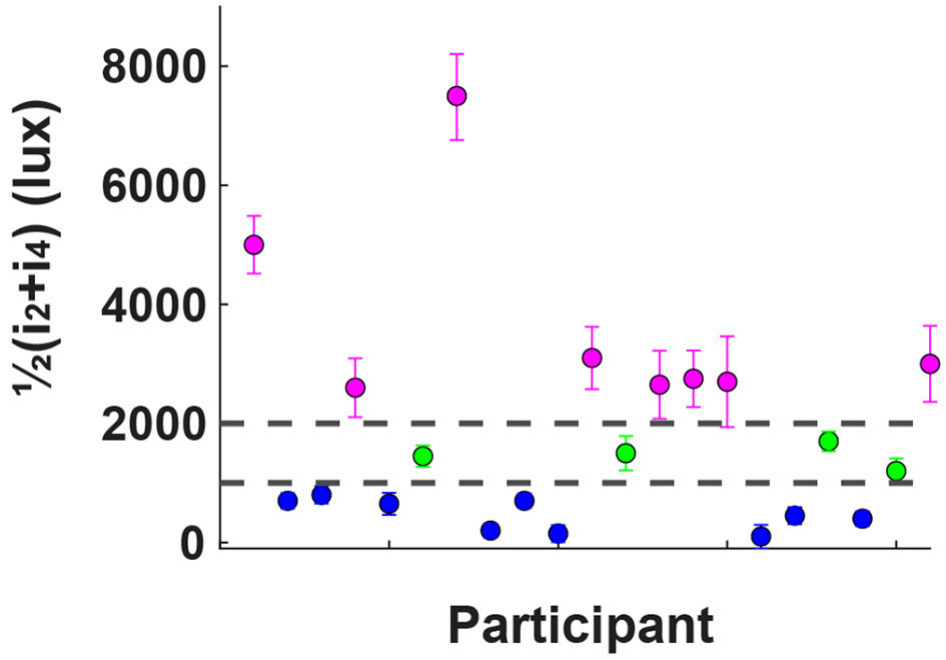
Results of the behavioral assessment, where participants indicated the light intensity at which discomfort begins (*i*_2_) and when it becomes unbearable (*i*_4_); the y-axis indicates the average of these two values for each participant. Based on previous research, two thresholds were established: one at 1,000 lux and another at 2,000 lux. Participants whose high discomfort threshold fell between these two values were labeled as “average sensitive” (shown in green). Those with thresholds below 1,000 lux were categorized as “hypersensitive” (blue), and those above 2,000 lux as “hyposensitive” (magenta). Only the non-average participants, 17 out of 21, proceeded to the subsequent EEG assessment.

The protocol consisted of two phases: a discomfort thresholds assessment without EEG recording, followed by an EEG recording phase. In the first phase, the dome emitted a series of exponentially increasing light intensities, starting at 45 Lux and reaching up to 10,255 Lux. Each flash had a duration of 1s, interleaved with 2 s of no light, as depicted in Figure 2A. Participants were instructed to press a button twice during the experiment, to indicate the extent of their discomfort level in response to the projected light intensity. The first button press occurred when the participant began to feel minor discomfort, establishing her/his individual low discomfort threshold *i*_2_. The second button press occurred when the light intensity became so strong that the participant found it difficult to keep her/his eyes opened, defining the high discomfort glare threshold *i*_4_. At the time of the second button press, the light intensity ramp stopped and the trial ended. To obtain a reliable, though subjective, estimate of *i*_2_ and *i*_4_ for each participant, the procedure was repeated four times, the two subjective thresholds were averaged and participants were ranked based on their 2 threshold values. We focused on participants who exhibited “extreme” sensitivity to light, that we called either hyposensitive or hypersensitive. Therefore, only participants whose average threshold did not fall between 1000 and 2000 lux (17 out of 21) proceeded to the next phase of the study, as shown in Figure 3. This threshold range, and hence the criterion to define hyper and hypo-sensitive individual, was chosen based on previous research (Marié et al., 2021).

The second phase of the experiment involved recording EEG neural activity in four experimental conditions corresponding to the exposure to four different light intensities: a no-discomfort light intensity *i*_1_= $\frac{i_{2}}{2}$, the low discomfort condition *i*_2_, a medium discomfort condition *i*_3_= $\frac{i_{2}+i_{4}}{2}$, and the high discomfort glare condition *i*_4_. Each condition included 50 trials, resulting in a total of 200 trials, with all conditions randomized. The experiment was divided into five sessions, of 40 randomized trials, with a 2 min break between sessions. Participants were instructed to fixate on the center of the dome, with their heads supported by a chin-rest. No tasks were performed during the trials. The dome emitted light flashes at a random intensity *i*_*k*_ for 1 s, followed by no-light intervals of randomly varying duration between 2 and 7 s, as illustrated in Figure 2B. The randomization of both time intervals and trial conditions was designed to prevent photo-receptor adaptation (Fain et al., 2001) and to reduce the onset of potential brain prediction mechanisms (Bar, 2007; Kveraga et al., 2007; Bar, 2011). Of the 17 subjects initially recorded, data from 16 were retained due to the poor data quality of one participant.

### 2.4 EEG data acquisition and statistical analysis

**Figure 4:**
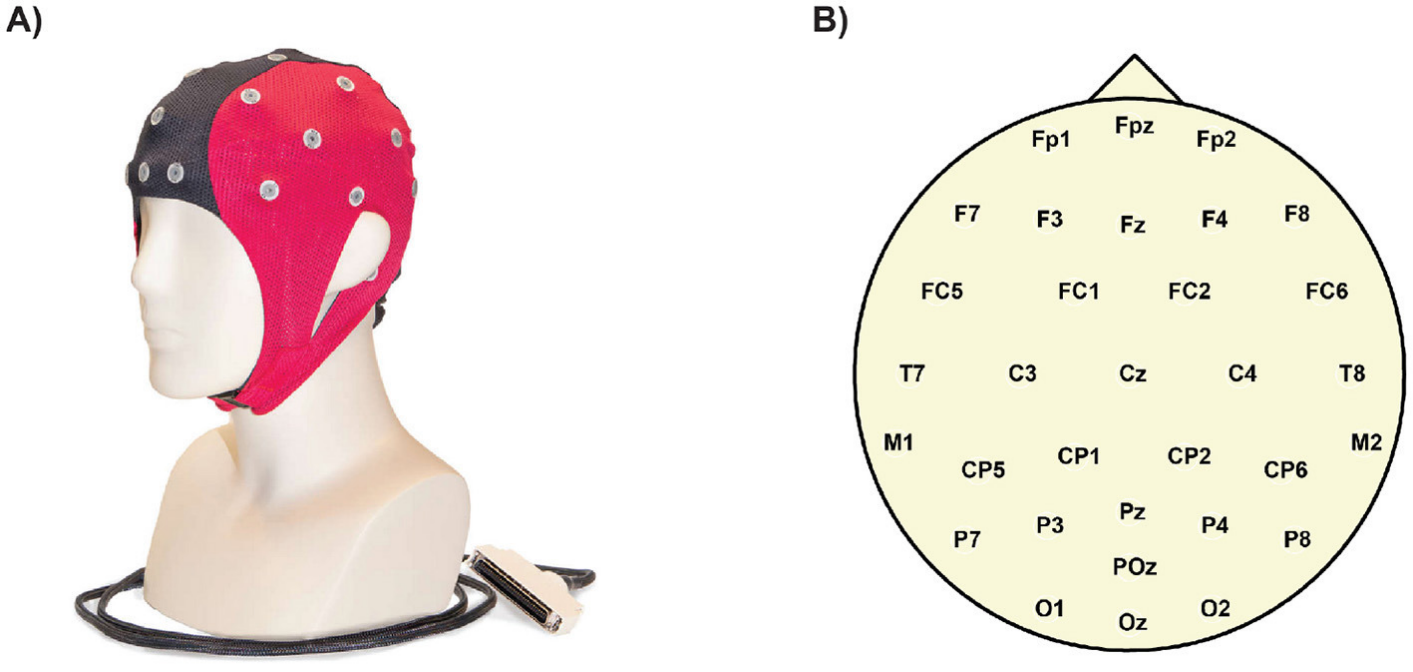
An EEG (electroencephalography) cap is a specialized headgear fitted with multiple electrodes that rest on the scalp. These electrodes detect and record the brain's electrical activity (brain waves). **(A)** EEG cap AntNeuro, 32 channels. **(B)** Ant Neuro Waveguard original electrodes layout, standard 10/20 system.

A 32-channel EEG cap Figure 4A with passive wet electrodes (Waveguard original, following the standard 10/20 system; ANT Neuro) was used, connected to an eego mylab amplifier with a sampling rate of 500 Hz (Figure 4B for electrode positioning). The EEG data were pre-processed using the EEGLAB toolbox in MATLAB (Delorme and Makeig, 2004). First, the raw data were filtered between 1 Hz and 45 Hz. Channels containing artifacts were identified and interpolated and then all channels were re-referenced to the mean. Independent component analysis (ICA) was performed to decompose the EEG signals into independent components, which were then labeled using the ICLabel algorithm. The components most likely to reflect brain activity were selected and used to reconstruct the data in the electrode space for further analysis.

The time-series data were segmented into epochs, each beginning 1 second before stimulus onset and ending 2 s after stimulus offset. This segmentation allowed for the extraction of Event-Related Potentials (ERPs), which reflect brain responses time-locked to the stimuli. ERPs were averaged across epochs and subjects to generate grand-averages (GA), thereby maximizing the signal-to-noise ratio. In addition to time-domain analysis, a Fourier transform was applied to each epoch to examine the different oscillation modes of neural activity.

To evaluate the significance of the observed effects, permutation-based statistical analyses were used (Maris and Oostenveld, 2007; Cohen, 2014), with statistical threshold (p-value) of 0.05. These tests assume that under the null hypothesis, the data are exchangeable, and any observed differences could have arisen by chance if group labels were randomly assigned. By recalculating the test statistic across numerous permutations of the data, an empirical distribution under the null hypothesis is generated, providing an unbiased assessment of whether the results were statistically significant.

The analysis spanned multiple dimensions, including temporal intervals, electrode sites, and spectral frequency bands, to capture the full scope of neural dynamics. To address the issue of multiple comparisons and prevent inflation of Type I error rates (Luck, 2014; Cohen, 2014), the False Discovery Rate (FDR) correction method was applied (Benjamini and Hochberg, 1995; Delorme and Makeig, 2004; Cohen, 2014; Luck, 2014). Unlike traditional corrections like the Bonferroni method, which control the family-wise error rate but can be overly conservative, the FDR method controls the expected proportion of false discoveries. This approach maintains statistical power, allowing for the identification of true positive findings while controlling the false discovery rate, making it more suitable for large datasets.

### 2.5 Verification of synchronization

**Figure 5:**
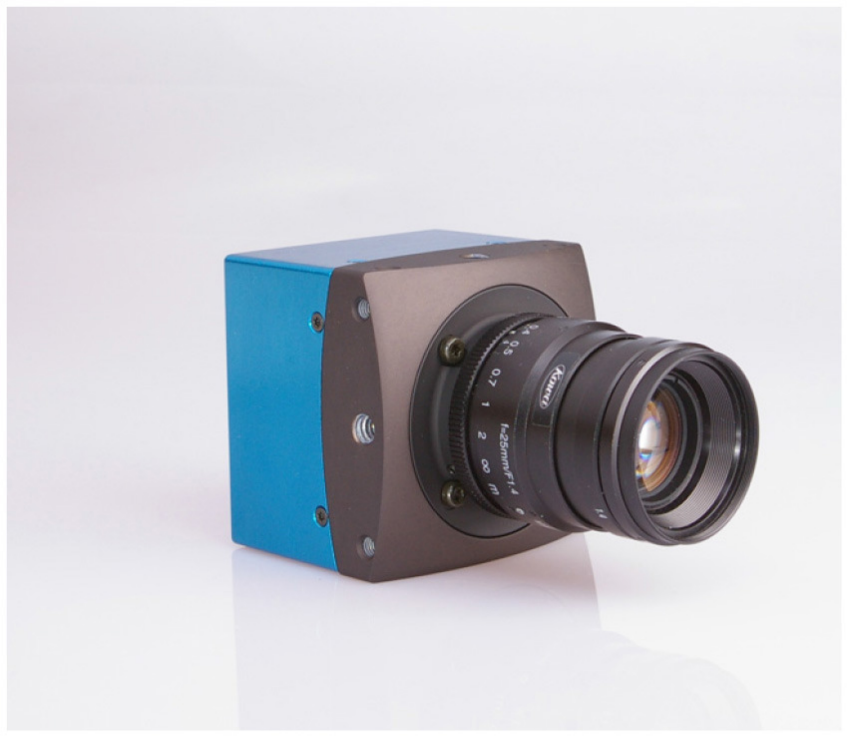
High-speed MIKROTRON Mini2 MGE-CM4 camera used to verify the synchronization between stimuls presentation and timestamps in neural recordings.

Precise synchronization of stimulus presentation with neural recordings is essential in neurophysiological studies, as even slight timing deviations can lead to significant misinterpretations of neural activity. To achieve exact alignment with EEG data, we utilized the MIKROTRON Mini2 MGE-CM4 high-speed camera to measure latencies in the light stimuli emitted by the coupole. Before initiating the experiments, calibration tests were conducted to confirm the synchronization between the coupole and EEG system by recording events with known time signatures. The MIKROTRON Mini2 MGE-CM4 (Figure 5), featuring a CMOS sensor and global shutter, provides a native resolution of 1,696 × 1,710 pixels and supports frame rates of up to 200,000 fps at reduced resolutions. This capability was vital for capturing fast events with microsecond-level precision. The camera's sensitivity range (400–900 nm) matched the visible spectrum of the coupole's light output. By adjusting the region of interest and synchronizing the camera's clock with the EEG system, we established a shared temporal framework for both visual and neural data collection.

## 3 Results

As illustrated in Figure 3, the behavioral assessment revealed significant individual variability in discomfort levels across the different light intensities tested. Based on these results, we divided the participants into two primary clusters, hypersensitive and hyposensitive groups, according to their individual responses to light, as described in the Methods section. This classification allowed us to proceed with the EEG analysis. EEG results are shown based on a general population analysis, as well as group comparisons. In the former case, we seek to identify the cortical response as a function of the different light intensities tested, i.e. *i*_1, 2, 3, 4_, disregarding the individual sensitivity to light. To the contrary, in the latter case we highlight the differences in cortical responses between hypo and hypersensitive subjects.

### 3.1 General population analysis

First, we analyzed the data from all 16 subjects collectively. It is important to reiterate that the value of *i*_*k*_ (where k refers to the different discomfort intensity levels) differs between subjects. This is because each *i*_*k*_ is derived from each individual's specific discomfort threshold, which is subjective and vary across participants. For instance, *i*_1_, i.e., the no-discomfort light intensity, is calculated as half of *i*_2_, i.e. the low discomfort threshold, which was determined individually for each participant in the discomfort glare assesment phase. As a result, each subject's set of *i*_*k*_ values is uniquely associated to a specific subject, reinforcing the personalized nature of this experiment and the inherent variability in light sensitivity across individuals.

**Figure 6:**
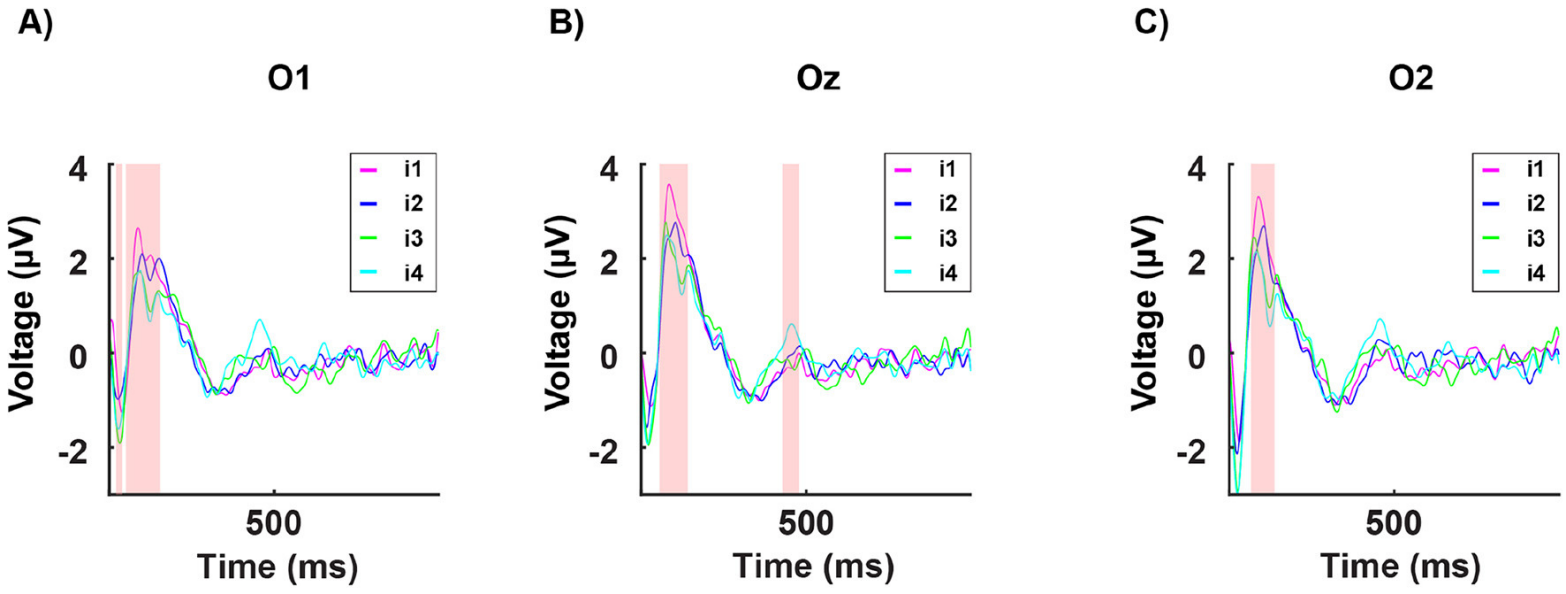
Grand average of the signal in time from stimulus onset (T = 0) for all 16 participants for a single occipital channel. Each curve indicates the neural response to the four subject-specific light intensities determined, for each individual, via the behavioral assessment: *i*_1_ (in magenta): no discomfort, *i*_2_ (in blue): light discomfort, *i*_3_ (in green): high discomfort, *i*_4_ (in cyan): unbearable discomfort. **(A)** shows data from the occipital channel O1, **(B)** from Oz, and **(C)** from O2. The red-shaded regions indicate significant differences in power between the four responses (for the Statistical Methods see EEG Data acquisition and statistical analysis).

**Figure 7:**
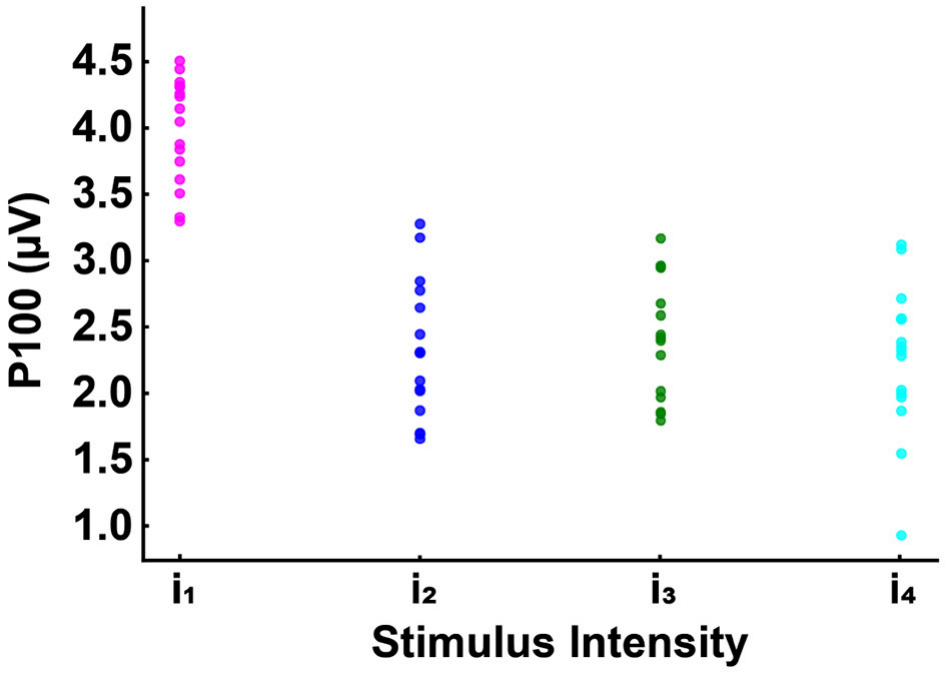
Grand averages of the P100 potentials at channel Oz in response to the four light intensities tested, with each participant's value represented by a dot: *i*_1_ (in magenta): no discomfort, *i*_2_ (in blue): light discomfort, *i*_3_ (in green): high discomfort, *i*_4_ (in cyan): unbearable discomfort. P100 potential in the no discomfort condition (*i*_1_) significantly different (*p* < 0.001) from the potential at the discomfort conditions (*i*_2_, *i*_3_, *i*_4_) for all subjects tested.

As expected from previous research (Bargary et al., 2015), we observed statistically significant findings in the occipital channels (O1, Oz, O2). Moreover, as shown in Figures 6 and 7, we found a significant difference between the amplitude of the P100 in the no-discomfort condition *i*_1_ in respect to the P100 elicited by the discomfort conditions *i*_2, 3, 4_.

**Figure 8:**
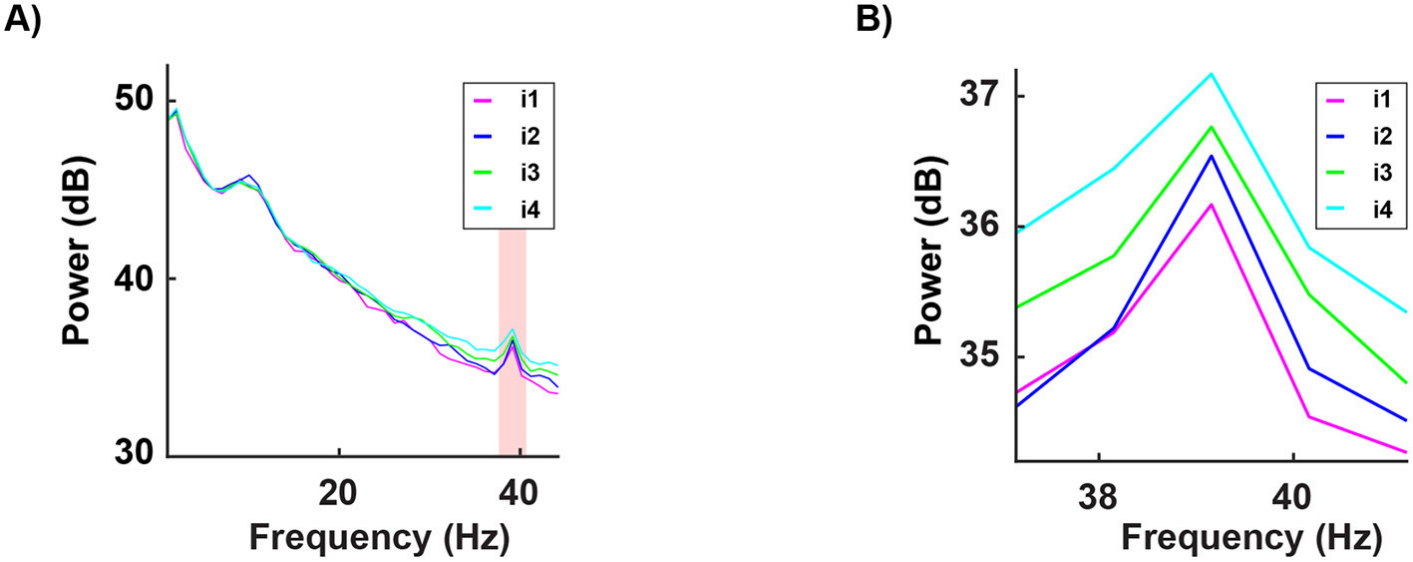
Grand average of the signal in frequency for all 16 participants and for a subset of channels (mean of O1, O2, and Oz). Each curve indicates the neural response to the four subject-specific light intensities determined, for each individual, via the behavioral assessment: *i*_1_ (in magenta): no discomfort, *i*_2_ (in blue): light discomfort, *i*_3_ (in green): high discomfort, *i*_4_ (in cyan): unbearable discomfort. Panel B provides a zoom-in of the spectrum between 37 and 41Hz, emphasizing the correlation between stimulus intensity and spectral energy, particularly around the 39 Hz peak. The red-shaded regions indicate significant differences in power between the four responses (for the Statistical Methods see EEG Data acquisition and statistical analysis). Pearson's correlation (at 39.3 Hz) between the conditions: *i*_1_ vs *i*_2_ *r* = 96, *i*_1_ vs *i*_3_ *r* = 95, *i*_1_ vs *i*_4_ *r* = 93, *i*_2_ vs *i*_3_ r = 97, *i*_2_ vs *i*_4_ *r* = 94, *i*_3_ vs *i*_4_ *r* = 92. All correlations were statistically significant (*p* < 0.001) and were observed in all 16 subjects tested. Importantly, the increased gamma activity may function as a protective response to alleviate the harmful effects of discomfort.

Additionally, in the occipital channels, we found a positive correlation between the discomfort of the stimulus and the power in the gamma frequency range, particularly around the 40 Hz peak, as illustrated in Figure 8; hence, the most disturbing light triggers the highest gamma oscillations; interestingly, intermediate light intensities are triggering less oscillations than the most disturbing level, but more than the lowest one, generating a clear correlation pattern between the extent of the cortical oscillations and the discomfort associated to light exposure.

### 3.2 Group comparisons

**Figure 9:**
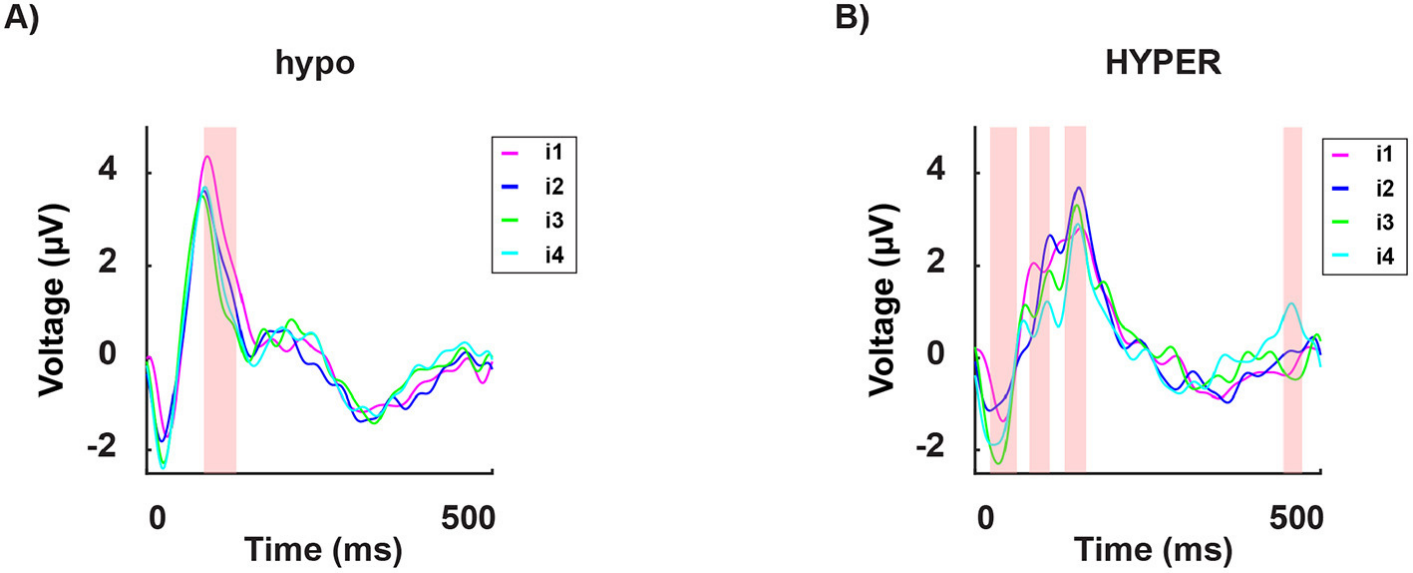
Grand average of the signal in time from stimulus onset (T = 0) for a subset of channels (mean of O1, O2, and Oz), for the hyposensitive group [**(A)**, 8 subjects] and the hypersensitive group [**(B)**, 8 subjects]. Each curve indicates the neural response to the four subject-specific light intensities determined, for each individual, via the behavioral assessment: *i*_1_ (in magenta): no discomfort, *i*_2_ (in blue): light discomfort, *i*_3_ (in green): high discomfort, *i*_4_ (in cyan): unbearable discomfort. The red-shaded regions indicate significant differences between the four responses.

Next, we divided participants into two groups: eight hypersensitive and eight hyposensitive to light (see the Methods section). Interestingly, in the hyposensitive group, the P100's amplitude to the less disturbing light intensity *i*_1_, was significantly higher than the responses to all other light intensities *i*_2, 3, 4_, which were indistinguishable as shown in Figure 9.

**Figure 10:**
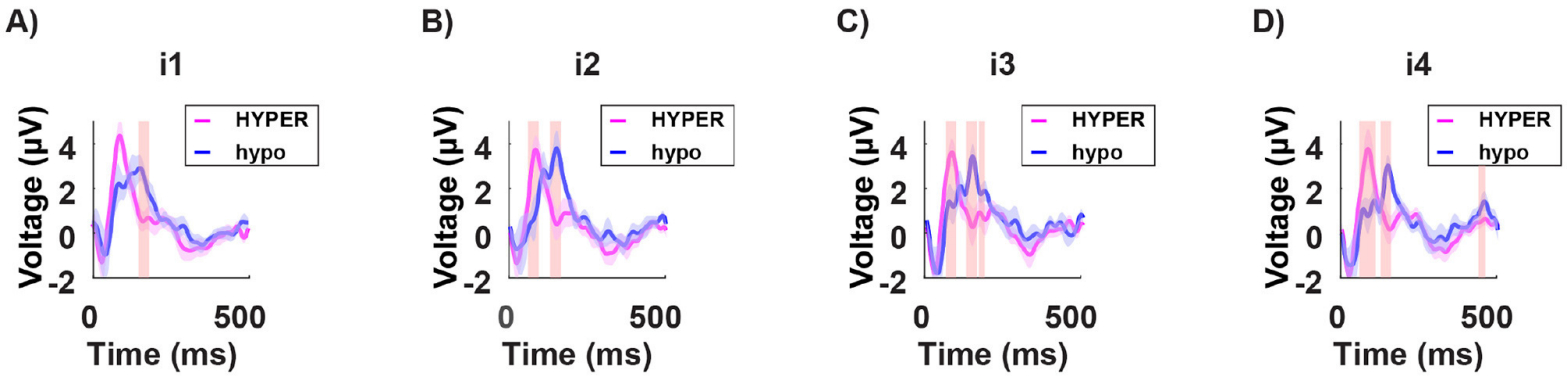
Grand average of the signal in time from stimulus onset (T = 0) for a subset of channels (mean of O1, O2, and Oz) in both the hypersensitive group (in magenta) and the hyposensitive group (in blue), in response to the four subject-specific light intensities: *i*_1_ no discomfort; *i*_2_ light discomfort; *i*_3_ high discomfort; *i*_4_ unbearable discomfort. The red-shaded regions indicate significant differences between the two groups. Across all conditions, the hypersensitive group consistently demonstrates shorter latencies for the P100 component. This may suggest a general overreaction system for the hypersensitive individuals, where stimulation leads to heightened reactivity.

Subsequently, we analyzed the neural response to each light intensity separately *i*_1, 2, 3, 4_, and compared it between the two groups. The hypersensitive group consistently exhibited shorter latencies in the P100 peak across all intensities (Figure 10).

**Figure 11:**
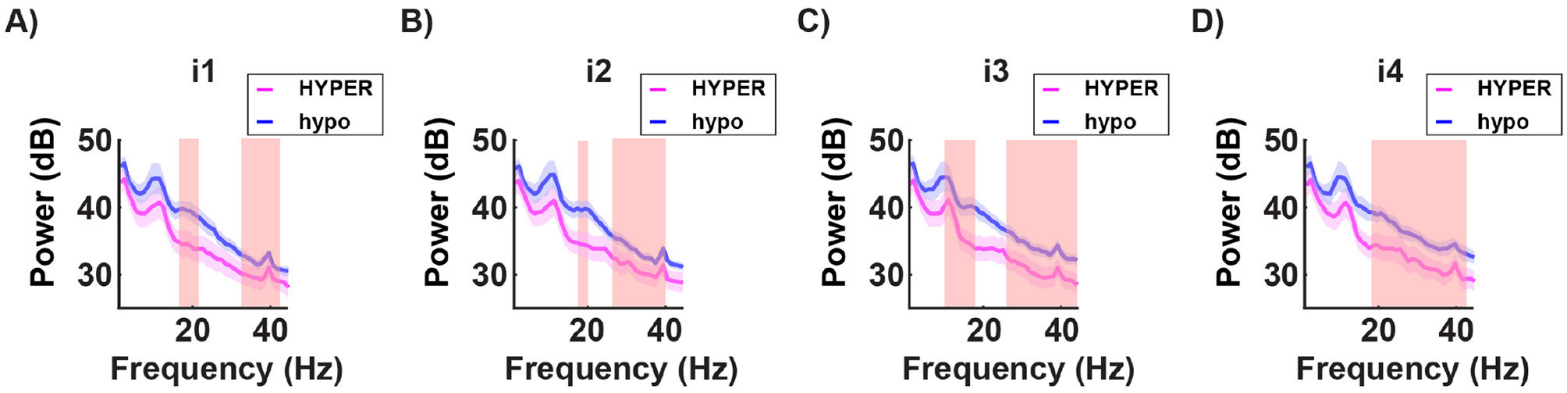
Grand average of the power spectra for a subset of channels (mean of Fp1, Fp2, and Fpz) in both the hypersensitive group (in magenta) and the hyposensitive group (in blue), in response to the four subject-specific light intensities: *i*_1_ no discomfort; *i*_2_ light discomfort; *i*_3_ high discomfort; *i*_4_ unbearable discomfort. The red-shaded regions indicate significant differences between the two groups. Across all conditions, the hyposensitive group consistently shows higher energy levels in the beta and gamma frequency bands. This suggests an underlying difference in stimulus processing between the two groups, with stronger power in the higher frequencies potentially associated with a more robust protective mechanism in the hyposensitive individuals.

In the spectral domain, significant differences in the energy power were found in the prefrontal channels (Fp1, Fpz, Fp2) across the beta and gamma bands. The hyposensitive group consistently showed higher energy levels for all light conditions tested (Figure 11).

To summarize, we found the following pattern of results:

**General population analysis**:

- significant difference of the neural response at P100 between no discomfort and discomfort conditions;
- significant correlation between the discomfort triggered by the light intensity and the power of the gamma band in occipital channels.

**Group comparisons**:

- while the hypersensitive group does not show a clear pattern of P100 activation, the hyposensitive group, shows a mostly invariant response to all the uncomfortable light intensities but a significantly stronger P100 potential;
- the hypersensitive group shows consistently shorter latencies of the P100 peak for all light intensities;
- the hyposensitive group consistently shows significantly higher energy levels in the beta and gamma bands in the prefrontal channels across all light intensities.

## 4 Discussion

This study, relying on the difference between the EEG activity of hypersensitive and hyposensitive individuals, proposes a new perspective on the neural mechanisms underlying light sensitivity.

When analyzing the data as a whole, disregarding the individual sensitivity to light, we found significant difference between the discomfort caused by high light intensities and the amplitude of the P100 response in occipital channels (O1, Oz, O2). Additionally, we found a positive correlation between discomfort and gamma-band power, specifically around 40 Hz. This significant difference in time and correlation in frequency may reflect an anomalous neuronal processing triggered by the exposure to an uncomfortable light stimulation (Bargary et al., 2015; Juricevic et al., 2010). Consistently, visual discomfort, often accompanied by headache or nausea (Boyce and Wilkins, 2018), has been proposed to be linked to an abnormal neuronal processing accompanied by a high metabolic demand (Fernandez and Wilkins, 2008; Juricevic et al., 2010). In other words, discomfort could be a homeostatic response to the excessive metabolic load on the system (Haigh et al., 2013; Wilkins et al., 1984). Our EEG analysis revealed a significant increase in the gamma band associated to the exposure to the more uncomfortable light intensities, suggesting the brain's effort to handle the overwhelming and uncomfortable sensory input. These findings align with existing frameworks highlighting the critical role of high-frequency bands in sensory processing (Buzsáki, 2006; Neuper and Pfurtscheller, 2001) and discomfort or pain-related mechanisms (Bassez et al., 2020; Li et al., 2016; Zhou et al., 2018; Gross et al., 2007; Wang et al., 2020). The involvement of the gamma band likely reflects neural gain control mechanisms, where increased sensory discomfort leads to stronger cortical excitation and stronger gamma oscillations. Similar patterns of time and frequency domain correlations have been observed in other sensory processing contexts, such as resonance phenomena in the visual cortex during flicker stimulation (Herrmann, 2001).

In the light of all these studies as well as our own results, we propose that the stronger gamma activity, may serve as a protective response to mitigate the harmful effects of discomfort. It may represent a neuroprotective mechanism, dispersing excessive sensory input across various neural pathways to prevent localized overstimulation and potential damage. This interpretation aligns with the views of Haigh and Wilkins (Haigh et al., 2013; Wilkins et al., 1984), who have emphasized the importance of protective responses in sensory processing under extreme stimulation.

When analyzing the data according to the individual sensitivity to light, we found that in hypersensitive individuals, the light intensity modulates the response in a non-sistematic way, making the results harder to interpret. In contrast, the hyposensitive group showed minimal variation in response across different light intensities. Furthermore, the hypersensitive group exhibited significantly shorter P100 latencies across all light intensities, highlighting an altered temporal dynamics in early visual processing. This could suggest an overreaction of the system being overstimulated and hence becoming hyper-reactive (in time) to new incoming stimuli: in other words, it would be like repeatedly poking an unhealed wound. The results in the frequency domain are completely consistent with the idea that discomfort induces higher-frequency oscillations as part of a protective mechanism. Interestingly, the hyposensitive group exhibits stronger power in higher-frequency bands, which at a first glance may appear incoherent. However, this enhanced neural activity could indicate a more efficient neuroprotective mechanism at play in the hyposensitive group compared to the hypersensitive group. In this context, the stronger gamma and high-frequency oscillations may reflect an adaptive strategy for mitigating sensory overload, suggesting that the neural circuitry of hyposensitive individuals is better equipped to manage extreme sensory input without experiencing significant discomfort. Coherently, in the hypersensitive group, the neuroprotective response may be less effective, potentially leading to heightened discomfort under similar circumstances.

Importantly, this study underscores the value of tailoring interventions for sensory sensitivity based on personalized, objective data. Traditional approaches to treating sensory sensitivity often rely on generalized protocols, which may not account for the unique neural response patterns of individuals. This study suggests that by focusing on the specific neural mechanisms underlying the individual brain activity, clinicians can develop more effective, customized interventions. This approach could significantly improve quality of life for those with extreme sensory sensitivity, providing more effective and sustainable management strategies.

## 5 Conclusions

The study's conclusions are derived from a detailed analysis of EEG data, focusing on both time-domain and frequency-domain aspects. In the time domain, the analysis of the P100 component revealed that the amplitude of the P100 response in occipital channels is significantly associated with the discomfort induced by light intensity. Moreover, when comparing individuals with different sensitivities, hypersensitive individuals exhibited considerably shorter P100 latencies. This shortened latency suggests that their visual systems respond more rapidly, and perhaps overly so, to incoming stimuli. Such a rapid response may reflect an overreaction or a deficiency in the control mechanisms that normally modulate sensory input processing. In parallel, the frequency-domain analysis centered on gamma-band activity provided complementary insights. Specifically, there was a clear positive correlation between the discomfort experienced by participants and the power of gamma oscillations around 40 Hz. This increase in gamma activity is interpreted as the brain's effort to manage the overwhelming sensory input, acting as a form of neural gain control. The heightened gamma oscillations appear to serve as a protective response, attempting to counterbalance excessive stimulation. When considering group differences, the results revealed that hyposensitive individuals, despite demonstrating stronger power in higher-frequency bands, seem to engage an adaptive, neuroprotective strategy that effectively disperses sensory load and prevents overstimulation. In contrast, hypersensitive individuals, who exhibit more erratic modulation of responses combined with the faster P100 latencies, may have a less efficient neuroprotective mechanism. This inefficiency could contribute to their heightened levels of discomfort.

Overall, the integration of findings from both the time-domain and frequency-domain analyses supports the conclusion that abnormal neuronal processing in the occipital lobe is linked to light-induced discomfort. The observed alterations in the time course, as evidenced by changes in the P100 component, alongside the increased gamma oscillations, provide evidence that the brain employs neuroprotective mechanisms to mitigate the effects of excessive sensory input. In individuals who are less sensitive to light, these mechanisms appear to function more effectively, potentially reducing the overall discomfort experienced during intense light stimulation.

## Data Availability

The raw data supporting the conclusions of this article will be made available by the authors, without undue reservation.
